# Models of longevity (Calorie Restriction and AC5 KO): Result of three bad hypotheses

**DOI:** 10.18632/aging.100495

**Published:** 2012-10-28

**Authors:** Stephen F. Vatner, Dorothy E. Vatner, Lin Yan

**Affiliations:** Cardiovascular Research Institute, Department of Cell Biology and Molecular Medicine, University of Medicine and Dentistry of New Jersey, New Jersey Medical School, Newark, NJ, 07103, USA

## First Incorrect Hypothesis

Our major interest for several decades has been cardiovascular regulation, the sympathetic nervous system, beta adrenergic receptor (BAR) regulation including that by the G protein, Gsα, and messenger, adenylyl cyclase (AC) [1]. AC has nine mammalian isoforms. However, there are only two major isoforms in the heart type 5 (AC5) and type 6 (AC 6). Our hypothesis was that if one of the two, AC5 was disrupted, i.e., knocked out (KO), there would be major effects on cardiovascular regulation. This was not observed [2]. In fact the regulation of cardiac rate and contractility remained largely intact, even with BAR stimulation with isoproterenol. However, we did find that the hearts of AC5 KO mice were protected from cardiac stress, e.g., in response to either chronic pressure overload or chronic catecholamine stimulation [3], both interventions which induce cardiomyopathy.

## Second Incorrect Hypothesis

In lower organisms there is a link between stress resistance and longevity [4]. Our next hypothesis, based on the cardiac stress resistance in the AC5 KO, was that this model would extend longevity. The error in that hypothesis is that mice, unlike humans, do not die from heart disease. Nonetheless, we did this study and found the median life span of the AC5 KO was a third longer than WT, and the AC5 KO mice are protected against the cardiomyopathy and osteoporosis through mechanisms involving the MEK-ERK pathway and resistance to oxidative stress [5].

## Third Incorrect Hypothesis

The most widely studied model of longevity is calorie restriction (CR) [6]. Our hypothesis was that combining these two models would produce a super longevity model. Accordingly, we placed AC5 KO mice on CR. Within a month we found that all the AC5 KO mice on CR had died. Accordingly, we had to change our hypothesis to include that the AC5 KO and CR models share similar protective and metabolic mechanisms, which could mediate longevity and health, but when superimposed are actually lethal.

## Similar Metabolic Profiles in AC5 KO and CR [7]

Both models protect against obesity, with one important exception. In both models the animals weigh less than controls. In the CR model the animals eat less, by design, whereas the AC5 KO mice eat more than WT, but weigh less. The adiposity index is decreased as well as the size of fat pads. Furthermore, both models exhibit improved insulin resistance and glucose tolerance and show increased ketone bodies in the serum and reduced liver glycogen stores.

## Similar Gene Regulation in AC5 KO and CR [7]

To test the new hypothesis that both models share similar mechanisms we examined cDNA microarrays, not only in the heart but also in the brain, liver and skeletal muscle. Remarkably, the gene density heatmaps showed that AC5 KO has a remarkable similarity to CR mice in all four tissues studied, with the best correlations in heart and skeletal muscle. Furthermore, highly significant gene ontology (GO) entries were found in brain, heart, skeletal muscle, and liver. The majority of commonly regulated genes involved sensory perception, mitochondrial function, G-protein coupled receptor activity as well as protein processing including degradation, transport, and post-translational modification, skeletal muscle function, metabolism, and chromatin modification and organization. We then looked for other gene correlations that could relate to aging. Our microarray data analysis demonstrated downregulation of the G-protein coupled receptor protein signaling pathway, particularly the olfactory receptors and upregulation of ApoD were the most significant pathways shared by AC5 KO and CR mice suggesting that these mechanisms may be involved in the regulation of longevity in AC5 KO and CR, as has been reported for longevity in *C. elegans* and *Drosophila melanogaster*. In addition, we found Sirt1 and FoxO were upregulated in AC5 KO. These results suggest that sirtuins and FoxO transcription factors are involved in common pathways shared by AC5 KO and CR in the regulation of longevity and stress resistance. In further support of this, we recently found that this latter mechanism is critical for mediating cardiomyopathy that develops with chronic catecholamine stress in AC5 transgenic mice with overexpressed AC5 in the heart (Lai L et al; submitted)

## Future Directions

We have found that AC5 KO and CR mice share many tissue-specific pathways in the regulation of longevity and stress resistance, with a unifying feature of altered regulation of metabolism, resulting in lower body weight [7]. The demonstration that two models of increased longevity and stress resistance share metabolic phenotypes and genotypes, and also with aging models from lower organisms, will provide direction for future research, with the goal of developing novel therapeutic approaches.

**Figure 1:**
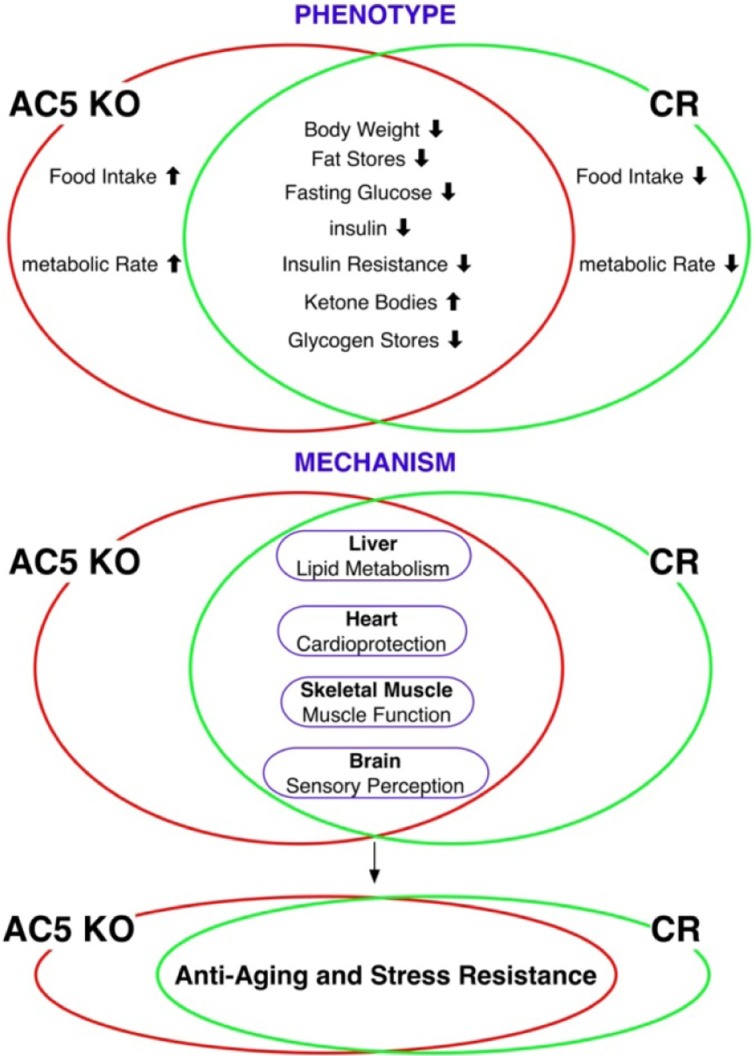
An outline for a unified theory of aging based on comparison of AC5 KO and CR models. (Reprinted with permission from Aging Cell [7]).
